# Fatigue states after cancer treatment occur both in association with, and independent of, mood disorder: a longitudinal study

**DOI:** 10.1186/1471-2407-6-240

**Published:** 2006-10-09

**Authors:** David Goldstein, Barbara Bennett, Michael Friedlander, Tracey Davenport, Ian Hickie, Andrew Lloyd

**Affiliations:** 1 Department of Medical Oncology, Prince of Wales Hospital, Sydney, Australia; 2 The Brain and Mind Research Institute, University of Sydney, Australia; 3 School of Medical Sciences, University of New South Wales, Sydney, Australia

## Abstract

**Background:**

Persistent fatigue is recognised as one of the most common, ongoing symptoms reported by patients following cancer treatment and may have profound effects on the quality of life. However, recent cross-sectional studies also highlight the close relationship between cancer related fatigue (CRF) and diagnoses of depression or anxiety disorder. There is currently limited information about the relationships between these conditions over time. We sought to examine the longitudinal relationships between fatigue and mood disorder in women treated with adjuvant therapy for early stage breast cancer.

**Methods:**

Women who had recently completed adjuvant therapy for Stage I or II breast cancer (n = 212) were sent a questionnaire with established case thresholds for clinically-significant fatigue and psychological disorder, as well as a questionnaire assessing disability. Potentially relevant variables linked to fatigue states, including age, treatment modality, menopausal status, and hematological indices were recorded. The illness outcomes were assessed over 48 months of follow-up.

**Results:**

The 176 women who responded to the questionnaire (84%) had a mean age of 55 (range 24–83) years and had completed adjuvant treatment on average 10 (range 4.7 – 16.3) months previously. Radiotherapy had been administered, either alone (50% of women) or in combination with chemotherapy (36%). Responses from 87 women (48%) indicated a significant fatigue state (termed here post-cancer fatigue; PCF), and from 59 women (33%) responses indicated significant psychological distress. Thirty-four women (19%) were cases of fatigue alone (i.e. unaccompanied by psychological disorder), whereas 52 (30%) were cases of both disorders. Multivariate analysis did not reveal any association between demographic, clinical or laboratory variables, and caseness for PCF. Self-reported functional disability was significantly associated with fatigue. Follow-up at 24, 36 and 48 months revealed high rates of ongoing PCF in conjunction with psychological distress, despite falling rates of psychological distress alone and fatigue alone.

**Conclusion:**

Post-cancer fatigue was prevalent and sustained on follow-up. Concurrent psychological disorder was evident in the majority, but not all, cases of PCF and tended to be sustained over time. Further prospective cohort studies to define the longitudinal co-morbid relationships between fatigue, mood disorder, and ongoing disability after cancer treatment are indicated.

## Background

Persistent fatigue is recognised as one of the most common, ongoing symptoms reported by patients following cancer treatment [1,2]. In those who have been otherwise successfully treated for malignant disorders, this problem may have profound effects on the quality of life [1]. Fatigue is a complex phenomenon, with physiological, psychological and behavioural dimensions [3]. When severe and persistent, fatigue states result in marked social, emotional and physical disability [4]. Fatigue is reported frequently in association with a broad range of medical conditions [5] and with a range of psychological disorders, notably major depression [6]. Previous research in primary care has indicated that prolonged fatigue states lasting one month or more occur in 25% of patients attending general practice, and that a third of these cases are not explained by other medical or psychological disorders [7].

In clinical oncology, fatigue is one of the most common unrelieved symptoms of cancer [8]. Such fatigue is an almost universal side effect experienced by patients during both radiotherapy [9-13] and chemotherapy [14-18], where the reported prevalence ranges between 70 and 100% [3,8,18]. Following completion of therapy, cross-sectional studies involving cancer patients with differing stages of disease, indicate that over 75% report fatigue as a significant ongoing symptom [19-21]. Application of the diagnostic criteria for cancer-related fatigue (CRF) indicates that between 14 and 30% of patients have ongoing CRF with associated disability when surveyed 1–5 years after completion of treatment, [21,22] well in excess of the rates in the general population [22]. Similarly, high prevalence rates of CRF have been reported in women receiving adjuvant therapy for breast cancer [13-18,23-32]. Recent prospective studies in both breast cancer and Hodgkin's disease survivors also consistently identified high rates of fatigue lasting months to years after treatment [33-36]. When the CRF persists well beyond the end of treatment (termed here post-cancer fatigue {PCF), it appears unlikely that the fatigue state can be simply accounted for by sustained consequences of the more obvious toxicities of adjuvant therapy such as anemia or menopause [27,35,37]. However, recent studies also highlight the close relationship between PCF and diagnoses of depression or anxiety disorder [24-26,38]. Accordingly, a cross-sectional cohort of women who had received adjuvant chemotherapy and/or radiotherapy for early stage breast cancer was recruited and then followed prospectively to determine the longitudinal relationships between these conditions. We hypothesized that the natural history of fatigue would vary according to the presence of other coexisting variables such as psychological symptoms and that the majority of cases would improve over time.

## Methods

Women (n = 212) who had attended the Medical Oncology Department at the Prince of Wales Hospital or the Cancer Care Center of St George Hospital, Sydney, Australia, for adjuvant treatment of surgically-resected stage I or stage II breast cancer were invited to complete a mail-out questionnaire, provided that they had completed adjuvant therapy in the preceding 12 months. The relevant institutional review boards approved the study and consent form which all patients signed.

### Screening questionnaires

The Somatic and Psychological Health Report (SPHERE) questionnaire was used to screen for clinically-significant fatigue states and mood disorders (see Additional file 1). This instrument was developed specifically to assess a wide range of somatic and psychological symptoms in medical and psychiatric settings [39] and has recently been used in assessing fatigue states in protracted fatigue following infectious illness [40] and in patients following adjuvant treatment for cancer [24]. The reliability and construct validity of the instrument in the identification of prolonged fatigue states have been demonstrated [41].

The Brief Disability Questionnaire (BDQ) [42] was used to assess social and occupational role impairment based on an 11-item self-rated tool. The scores on the BDQ have a possible range of 0–22 with each of 11 items scored 0,1,2 for the descriptors 'No, not at all', 'Yes, sometimes or a little', 'Yes, moderately or definitely'. The 11 items describe the capacity to participate in items ranging from "vigorous activity" to "eating, dressing, bathing". Self-reported disability on the BDQ has been demonstrated to consistently correlate with functional disability in the work role (including housework) [42].

### Clinical details

The treating oncologist provided clinical information, including details of the: surgical resection; tumor stage and nodal status; menopausal status prior to, and following, treatment; radiotherapy details (fields, dose, fractions and irradiated volume); and the chemotherapy details (drugs, doses, frequency, duration and on-treatment toxicity).

### Statistical analysis

Caseness for a clinically-significant fatigue state (i.e. PCF) was defined according to the previously validated cut-off scores on the SOMA subscale of the SPHERE [7,39]. Caseness for psychological disorder was similarly designated using the PSYCH subscale of the SPHERE [7,39]. Correlations between PCF, psychological and hematological variables were examined using multivariate modeling techniques. Logistic regression was used to seek predictive variables (and their associated relative risk) for PCF. All analyses were conducted using the SPSS for Windows version 10.0 [43].

## Results

### Demographics and baseline characteristics

One hundred and seventy six women (80% of the 212 surveyed) responded to the initial questionnaire. These women did not differ from those who did not respond in terms of age or treatment characteristics (data not shown). The respondents ranged in age from 26 to 85 years (mean 55 years). None of the women had disease progression evident at the time of the initial survey. Of the sample, 35% (n = 61) women were pre-menopausal, 12% (n = 21) peri-menopausal, and 52 % (n = 91) were post-menopausal prior to treatment. Of the former groups 51% (n = 42) had menopause induced by the adjuvant treatment. Fifty percent of the 176 women (n = 88) had received radiation therapy with or without subsequent tamoxifen; 36% (n = 63) had combined radiation and chemotherapy; and 13% (n = 23) had received chemotherapy with or without subsequent tamoxifen (Table 1).

**Figure 1:**
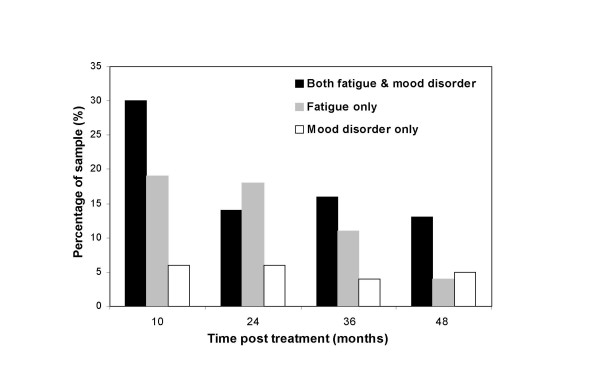
Longitudinal prevalence of fatigue and mood disorder in women followed after adjuvant treatment for early stage breast cancer (n = 176). Although the number of respondents decreased at 24, 36, and 48 months, the denominator was kept constant for these estimates.

Of the 176 respondents to the initial survey, 49% (n = 86) reported symptoms indicative of clinically-significant fatigue. Sixty-three women (36%) reported significant levels of anxiety or depression (see Figure 1) – of whom the majority (n = 53; 81%) were also cases of fatigue. Analysis of the responses given by 34 women (19%) indicated that they had prolonged fatigue as an isolated phenomenon (i.e. unaccompanied by psychological distress).

The mean disability score on the BDQ was significantly higher in the fatigue cases 9.2 (±5.7), than in the unaffected group (4.7 ± 4.7) (p < 0.0001), consistent with previous data indicating that the threshold for fatigue caseness on the SPHERE is associated with both patient and physician-rated functional impairment [39]. The women with fatigue with or without accompanying mood disorder reported significantly different patterns of functional status on individual items of the questionnaire. In particular, 32 of 80 women (40%) who were fatigue cases at enrolment reported 'moderate or definite' difficulty with walking up stairs compared with only 9 of 87 (10%) of those who were not fatigued (Chi-square 19.79 p < 0.001).

There was also no correlation with menopausal status pre-therapy, or with the advent of menopausal symptoms assessed at the time of the screening questionnaire. No relationship to treatment type, nodal status or tumor size was detected.

Logistic regression analysis (Table 2) incorporating the baseline clinical variables of treatment type, and menopausal status, as well as psychological symptom score at screening, identified psychological distress as the only significant risk factor for PCF (p < 0.0001).

### Longitudinal course

The 176 initial respondents were re-screened using the same instruments at 24 months post-treatment, when 137 (78%) of the original respondents returned completed questionnaires, at 36 months when 118 responded (67%), and finally at 48 months when 116 responded (66%). By the time of the 48-month post-treatment follow-up, 15 women of the original 176 who had responded (9%) were known to have relapsed or died from cancer recurrence and related complications. Although the falling rates of response make these data prone to selection bias at this late follow-up timepoint, of the respondents (41%) reported ongoing fatigue (vs. 49% at 10 months post treatment) with the proportion of women free of concurrent psychological distress remaining essentially unchanged at approximately 18% of the group as a whole. When these data were analysed more conservatively (by maintaining the denominator for the case rates at 176, that is, assuming non-respondents are asymptomatic) then 17% of the women were designated as having ongoing PCF, including 4% who had no associated psychological distress. There were 37 of 116 women at 48 months whose responses indicated significant levels of psychological disorder (32%) (vs. 36% at 10 months) and 27 (23%) with combined caseness (vs. 30% at 10 months). Again, conservative re-analyses of these data suggests that these figures would be 18% and 13% respectively (see Figure 1).

We also analysed the temporal fluctuations in each caseness category over the follow-up period between 10 and 36 months post treatment. Data for this period was available for 116 women, of whom 102 responded for all three timepoints and 14 did not respond at 24 months but did so at 36 months (Table 3). Of the 24 women in this group who reported fatigue initially, 13/24 (54%) reported resolution by 36 months post treatment, 6/24 (25%) had persistent caseness for fatigue, 1/24 (4%) had developed affective symptoms without fatigue, and 4/24 (16.7%) had developed mood disorder in combination with sustained fatigue.

Only 7/116 were identified with affective symptoms alone at the 10-month timepoint. Of this group, 3/7 (43%) had persistent mood disorder throughout follow-up, symptoms had resolved in 3/7 (43%), and 1/7 (14%) had newly acquired fatigue at the 36-month time point.

Both fatigue and affective symptoms were reported by 34/116 women at the 10-month timepoint. Of these women, 20/34 (59%) had persistent caseness for both components of the illness complex over the next two years, 10/34 (29%) subsequently reported fatigue only, and in 4/34 (12%) all symptoms resolved. No women from this group remained with affective symptoms only.

When first screened 51/116 women had no significant symptoms. Subsequently, 42/51 (82%) remained symptom free, 4/51 (8%) developed fatigue, 3/51 (6%) developed both fatigue and mood disorder and 2/51 (4%) developed affective symptoms alone.

Overall 15/116 (12.9%) women developed newly acquired fatigue symptoms alone after the first time point, 7/116 (6%) acquired symptoms of both fatigue and mood disorder, and only 3/116 (2.6%) acquired affective symptoms alone.

## Discussion

Consequent on the improvements in early identification [44] and intervention strategies for breast cancer there has been a marked increase in the number of women who undergo adjuvant therapy. The data reported here indicate that more than one third of these women experience prolonged morbidity characterized by fatigue after successful surgical and adjuvant treatments – termed here PCF. Previous studies have also highlighted the prevalence of PCF in women treated for breast cancer. For instance, Bower *et al *[36] in a study of 763 women who had completed treatment five to ten years previously, found that 34% of women reported fatigue up to 10 years post treatment. Andrykowski *et al *[45] used a case definition approach in a prospective study of 288 women who had received adjuvant treatment for breast cancer and identified a prevalence of fatigue of 26% at the completion of treatment. A prospective study [35] found that of 430 patients with a 3-year follow-up, 20% reported fatigue. There was no relationship with either chemotherapy regimen (high versus low dose) or mean hemoglobin level. Poor mental health (identified from Short-Form 36 – SF-36) was the best predictor of fatigue.

Fatigue is a common symptom accompanying both depressive and anxiety disorders [46]. In our data set the only significant predictor of fatigue was mood disorder (Table 2). However, studies in primary care as well as in oncology [8] and have identified prolonged fatigue states unaccompanied by psychiatric disorder [7,47]. A review of several studies of depression following adjuvant treatment in patients with breast cancer reported an overall prevalence of 26% [48]. The International Classification of Diseases (ICD-10) criteria for Cancer-related Fatigue specifically designates exclusion of patients with depression, as this disorder may provide an alternative explanation for the symptom set [49,50]. If applied to the questionnaire-based dataset reported here, 18% of women experienced PCF in the absence of psychological distress at 10 months post-completion of adjuvant treatment.

This is one of very few studies to examine the longitudinal evolution of PCF. This analysis indicated that the fatigue state was largely stable – that is the initial designation of fatigue was not likely to evolve into overt mood disorder on follow-up. This finding argues that PCF has independent determinants from mood disorder, and is consistent with similar findings in relation to CFS [47]. A similar pattern of gradual resolution over time was evident for both the fatigue and mood disturbance illness dimensions. However, the kinetics of resolution of the fatigue state was generally slower than that of psychological disorder. During extended follow-up, women with a combination of fatigue and psychological disorder ran a more persistent course than either those with psychological disorder alone or those with fatigue alone. Emergence of new cases of fatigue, either alone (15/116) or in combination with psychological disorder (7/116), was relatively rare. This finding argues that the genesis of both aspects of the illness complex was at the time of cancer treatment, rather than being attributable to subsequent events.

These data suggest that CRF may be reasonably separated into two temporally-defined groups. Firstly, a treatment-associated fatigue state with at least one clear etiologic factor – anemia [51]. The data for the negative influence of anemia on treatment-associated quality of life in general, and fatigue in particular, and improvement in these parameters following treatment with erythropoetin is well documented [51]. Secondly, cancer-related fatigue encompasses a group who has ongoing fatigue post-treatment – termed here PCF. In this latter group several studies have suggested that that anemia is unlikely to account for a significant proportion of cases of PCF [35,52]. Analysis of laboratory data from a subset of 47 subjects in the present report, revealed no association between lowered hemoglobin and the development of PCF (data not shown). Studies reported by Broeckel *et al *[29] and Bower *et al *[23] indicated that fatigue was more severe among patients with menopausal symptoms. Again, the data presented here showed no association between the fatigue state and induction of menopause. Similar findings indicating that fatigue states and menopausal symptoms are independent have recently been reported [53]. The relationship to the intensity of the original treatment is unclear, with some studies suggesting chemotherapy [23,30] and combination therapy [31], are more likely to be associated with protracted fatigue, and others finding no such association [32]. Taken overall it would appear that anemia and menopause do not provide a sufficient explanation for PCF.

These findings suggest that alternative hypotheses of the genesis of PCF will need to be explored. For instance, excessive production of pro-inflammatory cytokines that are known to be associated with induction of fatigue in the context of acute infection or immunotherapy, has been proposed [54]. Greenberg [9] correlated elevations in IL-1 levels with increasing fatigue scores in patients treated for prostate cancer with radiotherapy. Similarly, a cross-sectional study comparing 40 women with PCF five years post therapy for early stage breast cancer with a matched control group showed distinctive changes in cytokine profiles and T cell subsets [55]. An alternative endocrine hypothesis has been derived from recent data examining changes in the cortisol response to stressors in women with PCF suggesting that changes in the hypothalamic-pituitary-adrenal (HPA) axis may be relevant [56]. Such disturbances have also been implicated in relation to chronic fatigue syndrome [57].

## Conclusion

These preliminary findings emphasize the need for prospective data to document changes in relevant clinical and psychological parameters as well as putative biomarkers, in a longitudinal fashion to better understand the cause-and-effect relationships with illness outcomes in cancer survivors.

## Competing interests

The author(s) declare that they have no competing interests.

## Authors' contributions

DG, BB, AL, IH, MF and TD were all involved in designing the study. BB surveyed the patients, TD and BB analysed the data. All authors reviewed the data and were involved in final analysis and conclusions. DG wrote the first draft of the manuscript to which all authors subsequently contributed. All authors read and approved the final manuscript.

## Pre-publication history

The pre-publication history for this paper can be accessed here:



## Supplementary Material

Additional File 1 SPHERE Questionnaire. This is the SPHERE (Somatic and Psychological Health Report) self-report questionnaire used to screen for fatigue states. Click here for file

## Figures and Tables

**Table 1:** Demographic and clinical characteristics of women with and without post-cancer fatigue (PCF) after adjuvant treatment for breast cancer

**Patient groups; mean (SD) or percentage**			
	PCF (n = 98)	Non-fatigued (n = 77)	*P*
Age (years)	56 (11.5)	55 (11.5)	ns
Months since treatment completion	10 7 (6.1)	10.5 (5.6)	ns
**Surgical treatment (% of group)**			
Mastectomy	45.5	41.5	ns
Lumpectomy	54.5	58.5	ns
**Adjuvant treatment (% of group)**			
Chemotherapy	14.3	12.6	ns
Radiotherapy	45.5	53.7	ns
Combined chemotherapy and radiotherapy	40.3	33.7	ns
**Menopausal status at follow-up (% of group)**			
Pre-/peri-menopausal	34	39	ns
Postmenopausal	66	61	ns

**Table 2:** Logistic regression model – predicting post cancer fatigue at 10 months post treatment.

	**Beta co-efficient**	**Standard error**	**p value**	**O.R.(95% C.I.)**
	
**Variables**				
Mood disorder*	0.507	0.113	<0.0001	1.67 (1.33, 2.07)
Surgery type**	0.025	0.443	0.955	1.03 (0.43, 2.45)
Tumor size	0.305	0.546	0.577	1.37 (0.47, 3.95)
Menopause stage	0.037	0.240	0.877	1.04 (0.65, 1.66)
Chemotherapy (Y/N)	-0.063	0.595	0.915	0.94 (0.29, 3.01)
Radiotherapy (Y/N)	-0.559	0.692	0.419	0.57 (0.15, 2.22)
Tamoxifen (Y/N)	-0.006	0.470	0.990	0.99 (0.40, 2.50)
Constant	-0.723	1.151	0.530	0.49

C.I confidence interval; O.R. Odds ratio.

* Identified by SPHERE.

**Lumpectomy vs. mastectomy.

**Table 3:** Comparing changes in co-morbidity of fatigue and mood disorder over time in those who provided information at all three time points

Complete data available for three time points		10 months post-treatment (n = 116)	24 months post-treatment (n = 102)	36 months post treatment (n = 116)
Fatigue only n = 24	Unchanged	24/24	3/21	6/24
	Fatigue and mood disorder	N/A	2/21	4/24
	Mood disorder only	N/A	0/21	1/24
	Resolved	N/A	16/21	13/24
Mood disorder only n = 7	Unchanged	7/7	2/6	3/7
	Fatigue only	N/A	0/64	0/7
	Fatigue and mood disorder	N/A	1/6	1/7
	Resolved	N/A	2/6	3/7
Both fatigue and mood disorder n = 34	Unchanged	34/34	13/30	20/34
	Fatigue only	N/A	9/30	10/34
	Mood disorder only	N/A	3/30	0/34
	Resolved	N/A	5/30	4/34
Neither n = 51	Unchanged	51/51	30/45	38/49
	Fatigue only	N/A	10/45	4/51
	Fatigue and mood disorder	N/A	2/45	3/51
	Mood disorder only	N/A	3/45	2/51
