# Supplementary material for: Fatigue states after cancer treatment occur both in association with, and independent of, mood disorder: a longitudinal study
Source: BMC Cancer. 2006 Oct 9;6:240. doi: 10.1186/1471-2407-6-240 (PMC1613250; doi:10.1186/1471-2407-6-240)
Supplement: Additional File 1 — SPHERE Questionnaire. This is the SPHERE (Somatic and Psychological Health Report) self-report questionnaire used to screen for fatigue states. [file 1471-2407-6-240-S1.doc]

| Today’s date:____/_____/_____ | | | | | | | | | | | | | | |
| --- | --- | --- | --- | --- | --- | --- | --- | --- | --- | --- | --- | --- | --- | --- |
| SPHERE Questionnaire | | | | | | | | | | | | | | |
| **We would like to know about your general health. For ALL questions, please tick, cross or colour the circle that most closely matches your response. There are no right or wrong answers. Please answer ALL questions.** | | | | | | | | | | | | | | |
| Over **the past few weeks** have you been troubled by: | | | | | | | | | | | | | | |
|  | **Never or some of the time** | | | **A good part of the time** | | **Most of the time** |  | **Never or some of the time** | | | | **A good part of the time** | | **Most of the time** |
| 1. Headaches? | |  | |  |  | | 1. Feeling nervous or tense? | |  | | | |  |  |
| 1. Feeling irritable or cranky? | |  | |  |  | | 1. Feeling unhappy & depressed? | |  | | | |  |  |
| 1. Poor memory? | |  | |  |  | | 1. Feeling constantly under strain? | |  | | | |  |  |
| 1. Pains in your arms or legs? | |  | |  |  | | 1. Everything getting on top of you? | |  | | | |  |  |
| 1. Joint pain? | |  | |  |  | | 1. Being unable to overcome difficulties? | | | |  | |  |  |
| 1. Waking up tired? | |  | |  |  | | 1. Losing confidence? | |  | | | |  |  |
| 1. Rapidly changing moods? | |  | |  |  | | 1. Getting annoyed easily? | |  | | | |  |  |
| 1. Fainting spells? | |  | |  |  | | 1. Dizziness? | |  | | | |  |  |
| 1. Nausea? | |  | |  |  | | 1. Feeling tired after rest or relaxation? | |  | | | |  |  |
| 1. Arms or legs feeling heavy? | |  | |  |  | | 1. Feeling lost for the word? | |  | | | |  |  |
| 1. Weak muscles? | |  | |  |  | | 1. Diarrhoea or constipation? | | |  | | |  |  |
| 1. Muscle pain after activity? | |  | |  |  | | 1. Gas or bloating? | |  | | | |  |  |
| 1. Needing to sleep longer? | |  | |  |  | | 1. Fevers? | |  | | | |  |  |
| 1. Prolonged tiredness after activity? | | |  |  |  | | 1. Back pain? | | |  | | |  |  |
| 1. Poor sleep? | |  | |  |  | | 1. Sore throat? | |  | | | |  |  |
| 1. Poor concentration? | |  | |  |  | | 1. Numb or tingling sensations? | |  | | | |  |  |
| 1. Tired muscles after activity? | |  | |  |  | | 1. Feeling frustrated? | |  | | | |  |  |

SOMA scale (‘fatigue’): Items 12 to 17; PSYCH scale (‘mood disorder’): Items 18 to 23
